# A nomogram for predicting mortality risk within 30 days in sepsis patients admitted in the emergency department: A retrospective analysis

**DOI:** 10.1371/journal.pone.0296456

**Published:** 2024-01-25

**Authors:** Bin Wang, Jianping Chen, Xinling Pan, Bingzheng Xu, Jian Ouyang

**Affiliations:** 1 Department of Emergency, Affiliated Dongyang Hospital of Wenzhou Medical University, Jinhua City, China; 2 Department of Biomedical Sciences Laboratory, Affiliated Dongyang Hospital of Wenzhou Medical University, Dongyang, Jinhua City, China; Hokkaido University: Hokkaido Daigaku, JAPAN

## Abstract

**Objective:**

To establish and validate an individualized nomogram to predict mortality risk within 30 days in patients with sepsis from the emergency department.

**Methods:**

Data of 1205 sepsis patients who were admitted to the emergency department in a tertiary hospital between Jun 2013 and Sep 2021 were collected and divided into a training group and a validation group at a ratio of 7:3. The independent risk factors related to 30-day mortality were identified by univariate and multivariate analysis in the training group and used to construct the nomogram. The model was evaluated by receiver operating characteristic (ROC) curve, calibration chart and decision curve analysis. The model was validated in patients of the validation group and its performance was confirmed by comparing to other models based on SOFA score and machine learning methods.

**Results:**

The independent risk factors of 30-day mortality of sepsis patients included pro-brain natriuretic peptide, lactic acid, oxygenation index (PaO2/FiO2), mean arterial pressure, and hematocrit. The AUCs of the nomogram in the training and verification groups were 0.820 (95% CI: 0.780–0.860) and 0.849 (95% CI: 0.783–0.915), respectively, and the respective P-values of the calibration chart were 0.996 and 0.955. The DCA curves of both groups were above the two extreme curves, indicating high clinical efficacy. The AUC values were 0.847 for the model established by the random forest method and 0.835 for the model established by the stacking method. The AUCs of SOFA model in the model and validation groups were 0.761 and 0.753, respectively.

**Conclusion:**

The sepsis nomogram can predict the risk of death within 30 days in sepsis patients with high accuracy, which will be helpful for clinical decision-making.

## Introduction

Sepsis is a life-threatening condition resulting from an excessive immune response to infection, and is characterized by systemic inflammation that eventually culminates to multi-organ dysfunction [1, 2]. An estimated 11 million sepsis-related deaths occur worldwide every year, which accounts for 19.7% of all deaths. The global age-standardized mortality rate for sepsis is 148 deaths per 100,000 person-years [3]. Even in developed countries, the incidence rate of sepsis is 437 cases per 100,000 person-years and the hospital mortality rate due to sepsis is 17% [4]. The risk of death increases considerably once the sepsis progresses to septic shock [5]. Furthermore, the high fatality rate of sepsis leads to increased healthcare costs. Therefore, it is crucial to identify the sepsis patients with a high risk of death in order to improve prognosis through early interventions [6].

At present, the outcome of patients with sepsis is predicted using scoring systems based on various parameters. The “Acute Physiology and Chronic Health Evaluation II” or APACHEII score is widely used to assess the prognosis of critically ill sepsis patients [7]. However, studies show that the APACHE II score underestimates the risk of death in sepsis patients [8]. Similarly, the “Simplified Acute Physiology Score II” (SAPS II) has not been reliable in external validation studies [9, 10]. On the other hand, the sequential organ failure assessment or SOFA score is based on respiratory, cardiovascular, hepatic, coagulation, renal and neurological indices systems, and describes the development of multiple organ dysfunction, but is not effective for evaluating prognosis [11]. In addition, there is delay in prediction for the scoring systems mentioned above.

Several risk prediction models have been developed to evaluate the prognosis of sepsis patients, but there are still some deficiencies, the collinearity between continuous variables and logitp and the multi-collinearity between enrolled variables and the cross-interactions between enrolled variables have not evaluated before multivariate logistic regression analysis [12–14]. For machine learning models, it is hard to evaluate what variables were included and how much the enrolled variables contributed to the outcomes, leading to limited significance in practice [15, 16]. Although a few prediction models have been established to predict the death risk of sepsis patients, they are focused on the severe sepsis patients with shock or decreased blood pressure and they are accompanied by higher mortality [17–20]. In addition, the sepsis patients who are admitted in the emergency department usually suffer from emergent episode, consequently resulting uncertainty in prognosis. Thus, there have not been prediction model to evaluate the short-term prognosis of sepsis patients effectively who are admitted at emergency department. To this end, our aim was to establish a model to evaluate the mortality risk among the sepsis patients who are admitted at emergency department, displayed as a nomogram graph, which could provide a more understandable outcome measure and have been broadly adopted [21, 22].

## Materials and methods

### Study patient inclusion and exclusion

Sepsis patients who were admitted to the emergency department in Dongyang People’s Hospital from June 1, 2013, to September 1, 2021 were enrolled in this study (a retrospective analysis). The data collection was performed by extracting the information from clinical record database, which was constructed (by Hangzhou Le9 Healthcare Technology Co., Ltd) based on the patients’ clinical record after removing the personalized identifying information. The inclusion criteria were as follows: 1) definite diagnosis of an infection, and 2) an increase in SOFA score by 2 points or more [23]. Patients younger than 18 years of age, or with positive diagnosis of leukemia, lymphoma, chronic respiratory failure or end-stage tumor, uncertain prognosis indicators, missing data, incomplete treatment (given up treatment) or unknown infection were excluded.

This study was approved by the Ethics Committee of Dongyang People’s Hospital (NO. 2021-YX-093). The written consents of the patients or their families were obtained. Personal information was completely deleted from the data. This study was conducted in accordance with the principles of the Helsinki Declaration and its amendments.

### Variable collection

The basic demographic information (gender and age), Glasgow coma score (GCS), and the mean arterial pressure (MAP) were obtained from nursing record. The hematological indices at the time of admission such as oxygenation index (PaO2/FiO2), C reactive protein (CRP), creatinine (Cr), emergency procalcitonin (PCT), activated partial thromboplastin time (APTT), prothrombin time (PT), albumin, pro-brain natriuretic peptide (pro-BNP), lactic acid (LAC), hematocrit (HCT), platelet (PLT), white blood cell (WBC) count, total bilirubin (T-bil), sodium (Na), were measured and recorded in the clinical record within 24h of admission. The currently used international common units were used in this study.

### Data processing and selection of variables

Due to no linearity with logitp or cross-interactions between each other or multicollinearity among the enrolled variables, some variables were transformed into categorical variables.The patients were randomly divided into the training and validation groups at the ratio of 7:3 using the createDataPartition function in R statistical package and baseline characteristics between these two groups were analyzed. Univariate analysis was performed using the twogrps function of the CBCgrps package to identify the significant risk factors of mortality in training groups [24]. Multiple-collinearity among the variables and the significant variables were identified using variance inflation factor (VIFs) values. Variables had no cross-interactions each other (P > 0.05), were linear with logitp and without multiple collinearities (VIFs<10) were further included in the multivariate regression analyses and stepwise regression analysis. The independent risk factors were then used to establish the predictive logistic regression model and a nomogram graph was drawn to visually evaluate how much they contributed to the mortality risk [25].

### Evaluating the performance of established predict model

The discriminatory power of the model was analyzed by plotting receiver operating characteristic (ROC) curve, and the area under the curve (AUC) was calculated. AUC > 0.75 indicates good discrimination ability [26]. The cutoff value was determined based on the maximal Youden indexes in the ROC analysis, along with the sensitivity, specificity, the prediction accuracy, negative predictive value (NPV) and positive predictive value (PPV). A calibration chart was used to evaluate the goodness of fit, with a *P* value greater than 0.05 suggesting an adequate goodness of fit [27]. The decision curve analysis (DCA) was plotted to assess the clinical validity of the model. The distance of the model curve from the “All” and “None’ reference curves is indicative of clinical significance [28, 29]. Then, the established model was validated on patients in the validation group.

### Comparing to other prediction methods

Subsequently, random forests and stacking methods were employed to establish a model in the training group, the discrimination power of the models was compared to nomogram prediction model in the validation group by Delong test. Moreover, models based on the SOFA score were conducted in training group, the AUCs of the models were compared to nomogram prediction model in both training group and validation group.

### Statistical analysis

Variables with normal distribution were expressed as mean ± standard deviation, and data that were not normally distributed were expressed as median with quartiles. The categorical variables were displayed as counts with percentages. The significances between two groups were analyzed by twogrps function in CBCgrps packages. Other packages included caret, CBCgrps, tidyverse, car, rms, foreign, DataExplorer, boot, gbm, caretEnsemble, C50, xgboost, randomForest, mlr, reportROC.

## Results

### Baseline characteristics of the training population and validation population

A total of 2257 patients with sepsis from the emergency department were initially recruited (S1 Fig), of which 1052 patients were excluded due to age below 18 years (n = 75), unknown infections (n = 356), incomplete treatment (n = 32), lack of prognostic information (n = 125), comorbidities such as leukemia and chronic respiratory failure (n = 155), and missing data (n = 309). Finally, 1205 patients were included that were randomly divided into the training group with 844 cases and 139 deaths, and the verification group with 361 cases and 52 deaths. Accordingly, the mortality was calculated to be 16.5%. All baseline characteristics of the training population and validation population are shown in Table 1. There were no significant differences in the baseline characteristics between the training population and validation population (all p>0.05).

**Table 1:** Baseline characteristics of the training group and validation group^a^.

Variables^b^	Total (n = 1205)	Validation (n = 361)	training (n = 844)	p
Gender, n (%)				0.57
female	467 (39)	135 (37)	332 (39)	
male	738 (61)	226 (63)	512 (61)	
Aptt (s)	46.6 (41.6, 52.9)	46.4 (40.8, 52.6)	46.8 (41.9, 53.12)	0.403
PaO2/FiO2, n (%)				0.929
>300 (mmhg)	582 (48)	177 (49)	405 (48)	
<200 (mmhg)	227 (19)	66 (18)	161 (19)	
201–300 (mmhg)	396 (33)	118 (33)	278 (33)	
White blood cell (*10^9^/L)	10.43 (6.83, 15.01)	10.54 (6.79, 14.63)	10.39 (6.95, 15.03)	0.917
Creatinine, n (%)				0.651
<125 (umol/L)	721 (60)	219 (61)	502 (59)	
125–350 (umol/L)	416 (35)	125 (35)	291 (34)	
>350 (umol/L)	68 (6)	17 (5)	51 (6)	
Albumin, n (%)				0.418
>35 (g/L)	82 (7)	29 (8)	53 (6)	
<25 (g/L)	311 (26)	87 (24)	224 (27)	
25–35 (g/L)	812 (67)	245 (68)	567 (67)	
Map, n (%)				0.37
60–90 (mmhg)	687 (57)	214 (59)	473 (56)	
<60 (mmhg)	49 (4)	11 (3)	38 (5)	
>90 (mmhg)	469 (39)	136 (38)	333 (39)	
Hematocrit, n (%)				0.888
0.3–0.4	638 (53)	191 (53)	447 (53)	
<0.3	480 (40)	142 (39)	338 (40)	
>0.4	87 (7)	28 (8)	59 (7)	
Pct (ng/ml)	15.21 (3.12, 51.69)	15.41 (2.72, 49.47)	15.04 (3.27, 52.73)	0.892
Crp (mg/L)	170.6 (116.6, 200)	176.5 (117.85, 200)	169.3 (116.48, 200)	0.294
Sodium (mmol/L)	141.2 (138.2, 144.3)	141.1 (138.2, 144)	141.2 (138.2, 144.3)	0.781
Platelet, n (%)				0.436
101–300 (*10^9^/L)	704 (58)	221 (61)	483 (57)	
<100 (*10^9^/L)	443 (37)	124 (34)	319 (38)	
>300 (*10^9^/L)	58 (5)	16 (4)	42 (5)	
Pro.bnp, n (%)				0.751
<500 (pg/ml)	184 (15)	59 (16)	125 (15)	
500–15000 (pg/ml)	878 (73)	258 (71)	620 (73)	
>15000 (pg/ml)	143 (12)	44 (12)	99 (12)	
Lactic acid, n (%)				0.59
<4 (mmol/L)	860 (71)	265 (73)	595 (70)	
4–10 (mmol/L)	285 (24)	79 (22)	206 (24)	
>10 (mmol/L)	60 (5)	17 (5)	43 (5)	
Age (years)	75 (64, 83)	76 (64, 84)	75 (64, 83)	0.154
T-bil ((umol/L))	11.5 (7.7, 18.1)	11.1 (7.6, 17.6)	11.7 (7.8, 18.5)	0.228
Pt	15.6 (14.4, 17.3)	15.4 (14.4, 17)	15.6 (14.4, 17.53)	0.279
GCS	15 (14, 15)	15 (14, 15)	15 (14, 15)	0.783

Callout: a, Continuous variables are described by means and quarterbacks. Categories varies are analyzed by χ2 test and continuous variables are analyzed by Wilcoxon rank sum test; b, first examination index following admission.

Aptt, activated partial thromboplastin time; map, mean arterial pressure; pct, emergency procalcitonin; crp, high sensitivity–C reactive protein; pro.bnp, pro-brain natriuretic peptide; T-bil, total bilirubin; pt, prothrombin time; GCS, Glasgow coma scale score.

### Identification of variables associated with mortality risk in sepsis patients

In the training group, univariate analysis showed that 7 indices, including MAP, creatinine, oxygenation index, albumin, hematocrit, Pro.bnp, lactic acid were significantly correlated to prognosis (p<0.001) (Table 2).

**Table 2:** Univariate analysis between survivors and no survivors in training group^a^.

Variables^b^	Total (n = 844)	Survival (n = 705)	No Survival (n = 139)	p
Gender, n (%)				0.081
female	512 (61)	418 (59)	94 (68)	
male	332 (39)	287 (41)	45 (32)	
Aptt(s)	46.8 (41.9, 53.12)	46.3 (41.8, 52.7)	49.1 (42.8, 55.05)	0.014
PaO2/FiO2, n (%)				< 0.001
>300 (mmhg)	405 (48)	366 (52)	39 (28)	
<200 (mmhg)	161 (19)	106 (15)	55 (40)	
201–300 (mmhg)	278 (33)	233 (33)	45 (32)	
White blood cell (*10^9^/L)	10.39 (6.95, 15.03)	10.02 (6.96, 14.72)	12.55 (7.14, 17.72)	0.039
Creatinine, n (%)				< 0.001
<125 (umol/L)	502 (59)	444 (63)	58 (42)	
125–350 (umol/L)	291 (34)	228 (32)	63 (45)	
>350 (umol/L)	51 (6)	33 (5)	18 (13)	
Albumin, n (%)				< 0.001
>35 (g/L)	53 (6)	44 (6)	9 (6)	
<25 (g/L)	224 (27)	166 (24)	58 (42)	
25–35 (g/L)	567 (67)	495 (70)	72 (52)	
Map, n (%)				< 0.001
60–90 (mmhg)	473 (56)	412 (58)	61 (44)	
<60 (mmhg)	38 (5)	5 (1)	33 (24)	
>90 (mmhg)	333 (39)	288 (41)	45 (32)	
Hematocrit, n (%)				< 0.001
0.3–0.4	447 (53)	400 (57)	47 (34)	
<0.3	338 (40)	257 (36)	81 (58)	
>0.4	59 (7)	48 (7)	11 (8)	
Pct(ng/ml)	15.04 (3.27, 52.73)	14.96 (3.12, 48.95)	15.3 (4, 66.74)	0.091
Crp (mg/L)	169.3 (116.48, 200)	169.5 (120.04, 200)	164.5 (83.47, 200)	0.446
Sodium (mmol/L)	141.2 (138.2, 144.3)	141.3 (138.4, 144.1)	140.6 (136.75, 145.75)	0.817
Platelet, n (%)				0.003
101–300 (*10^9^/L)	483 (57)	421 (60)	62 (45)	
<100 (*10^9^/L)	319 (38)	249 (35)	70 (50)	
>300 (*10^9^/L)	42 (5)	35 (5)	7 (5)	
Pro.bnp, n (%)				< 0.001
<500 (pg/ml)	125 (15)	120 (17)	5 (4)	
500–15000 (pg/ml)	620 (73)	517 (73)	103 (74)	
>15000 (pg/ml)	99 (12)	68 (10)	31 (22)	
Lactic acid, n (%)				< 0.001
<4 (mmol/L)	595 (70)	542 (77)	53 (38)	
4–10 (mmol/L)	206 (24)	150 (21)	56 (40)	
>10 (mmol/L)	43 (5)	13 (2)	30 (22)	
Age (years)	75 (64, 83)	74 (63, 82)	78 (69, 85)	0.002
T.bil (umol/L)	11.7 (7.8, 18.5)	11.7 (7.9, 18.5)	12 (7.45, 18.1)	0.96
Pt (s)	15.6 (14.4, 17.53)	15.6 (14.3, 17.2)	16.1 (14.55, 18.45)	0.009
GCS	15 (14, 15)	15 (15, 15)	15 (14, 15)	0.008

Callout: a, Continuous variables are described by means and quarterbacks. Categories varies are analyzed by χ2 test and continuous variables are analyzed by Wilcoxon rank sum test;b, first examination index following admission.

Aptt, activated partial thromboplastin time; map, mean arterial pressure; pct, emergency procalcitonin; crp, high sensitivity–C reactive protein; pro.bnp, pro-brain natriuretic peptide; T-bil, total bilirubin; pt, prothrombin time; GCS, Glasgow coma scale score.

None of the significant variables showed obvious cross-interactions and multiple collinearity (S1 and S2 Tables), and they were all categorical variables. Multivariate logistics analysis further indicated that pro-bnp, lactic acid, PaO2/FiO2, map, and hematocrit were significantly associated with death (p<0.05; Table 3). Finally, stepwise regression analysis identified all of the above variables as independent risk factors related to the death of sepsis patients within 30 days (p<0.05; Table 3).

**Table 3:** Multivariable logistic and stepwise regression analysis of involved variables.

Variables^a^	Multivariable logistic regression		Stepwise regression	
	OR (95%CI)	P	OR (95%CI)	P
(PaO2/FiO2) <200 (mmhg)	3.568(2.040–6.284)	<0.001	3.447(1.975–6.051)	<0.001
(PaO2/FiO2)201-300 (mmhg)	1.551(0.909–2.650)	0.107	1.549(0.910–2.640)	0.106
Creatinine:125–350 (umol/L)	1.171(0.713–1.910)	0.530	NA	NA
Creatinine>350 (umol/L)	2.261(0.973–5.040)	0.051	NA	NA
Albumin<25(g/L)	1.496(0.516–4.987)	0.483	1.499(0.523–4.953)	0.476
Albumin:25-35(g/L)	0.714(0.256–2.305)	0.544	0.698(0.252–2.235)	0.514
Map<60(mmhg)	26.505(9.402–88.205)	<0.001	26.518(9.404–89.209)	<0.001
Map>90(mmhg)	1.050(0.659–1.664)	0.835	1.046(0.658–1.653)	0.849
Hematocrit<0.3	2.383(1.479–3.886)	<0.001	2.431(1.513–3.957)	<0.001
Hematocrit>0.4	1.939(0.767–4.525)	0.141	1.864(0.737–4.349)	0.166
Pro.bnp:500–15000 (pg/ml)	3.490(1.355–11.345)	0.019	3.367(1.447–11.938)	0.013
Pro.bnp>15000 (pg/ml)	5.089(1.674–18.467)	0.007	6.468(2.226–22.722)	0.001
Lactic acid:4–10 (mmol/L)	2.584(1.585–4.203)	<0.001	2.544(1.572–4.106)	<0.001
Lactic acid >10 (mmol/L)	10.103(4.483–23.428)	<0.001	10.759(4.800–24.837)	<0.001

Callout: a, first examination index following admission.

Map, mean arterial pressure; pro.bnp, pro-brain natriuretic peptide.

### Establishment and validation of the predictive nomogram for 30-day mortality

**Fig 1:**
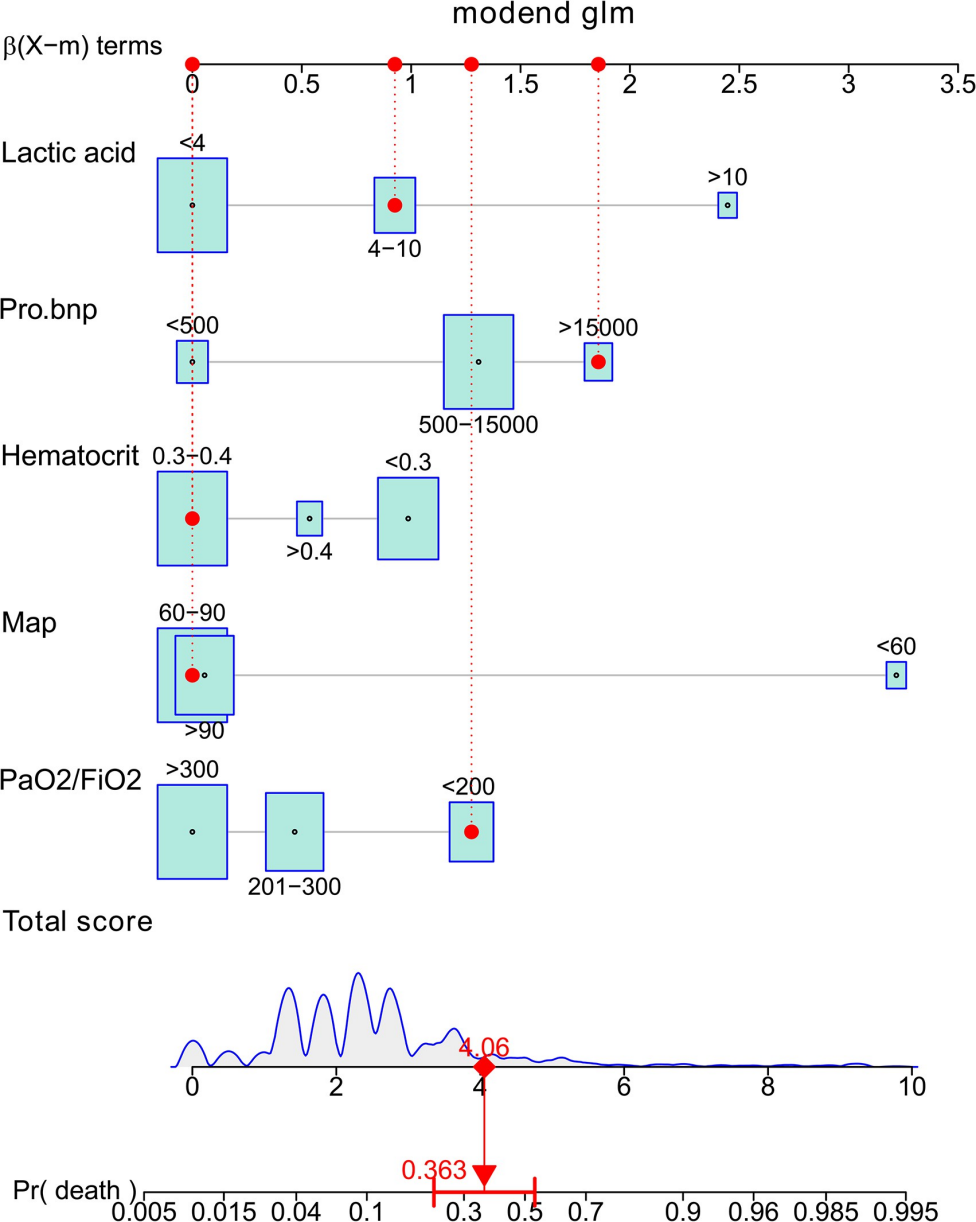
Nomogram graph established in this study. Map, mean arterial pressure; pro.bnp, pro-brain natriuretic peptide.

The independent risk factors of 30-day mortality were used to establish prediction model and was displayed as a nomogram (Fig 1). The score of each variable was calculated after matching to upper score line, and the individual scores were summed up to obtain the total score. Using the lower score lines and parallel prediction line, the total score was matched to the death risk.

### ROC curves, calibration belts and DCA curves of established model in the training group and the validation group

**Fig 2:**
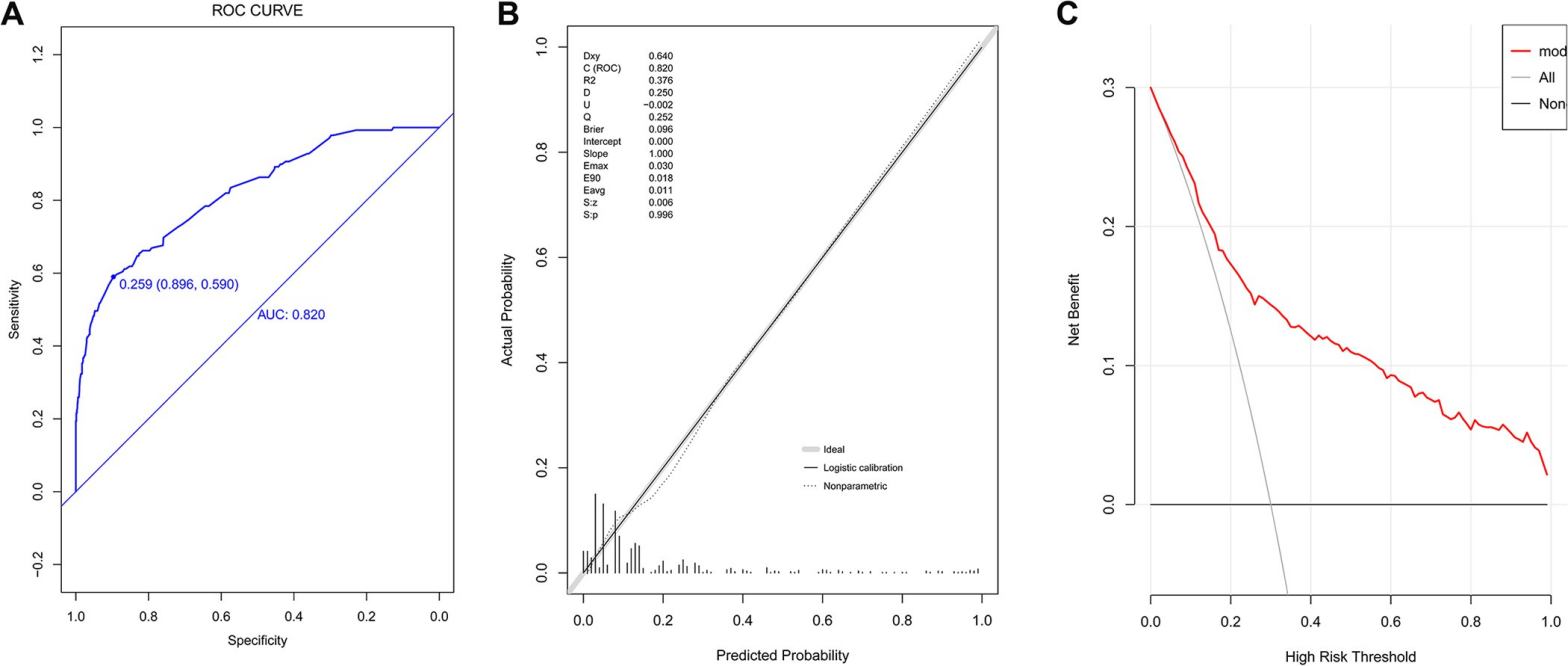
Evaluation of prediction model in the training population. A, ROC curve; B, calibration chart; C, DCA curve.

The discriminatory power of the nomogram was assessed by ROC analysis (Fig 2A). The AUC value of the nomogram in the training group was 0.820 (95% CI: 0.780–0.860), which is indicative of good discrimination ability. The cutoff value was 0.259, with sensitivity 89.7% (95% CI:87.2%-91.9%) and specificity 59.9% (95% CI:50.4%-66.9%). The prediction accuracy was 0.846 (95% CI:84.6%-84.6%), with a PPV of 0.529 (95% CI:45.0%-60.8%) and a NPV of 0.917 (95% CI:89.7%-93.8%).

The calibration chart was used to assess the goodness of fit, and the p-value, R^2^ and slope in the training group were 0.996, 0.376 and 1 respectively, suggesting good fit (Fig 2B). Finally, the DCA curves of the nomogram in the training group (Fig 2C) were above the two extreme curves, indicating good clinical efficacy.

**Fig 3:**
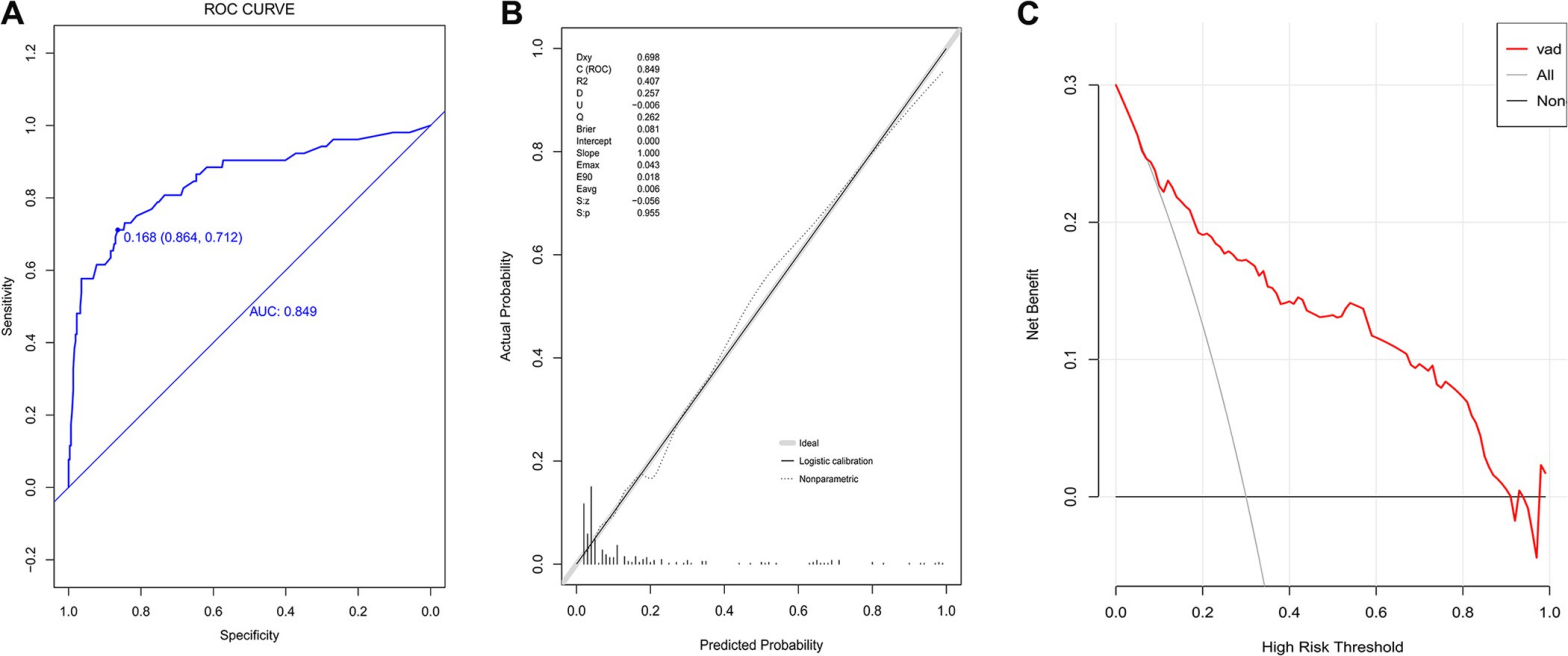
Evaluation of prediction model in the validation population. A, ROC curve; B, calibration chart; C, DCA curve.

The prediction model was further validated in the validation group. As shown in Fig 3A, the AUC of the model in the validation group was 0.849 (95% CI: 0.783–0.914), comparable to that in the training group. The prediction accuracy was 0.842 (95% CI:84.1%-84.3%), with a PPV of 0.468 (95% CI:35.8%-57.8%) and a NPV of 0.947 (95% CI:92.1%-97.3%).

The calibration chart (p = 0.955, R2–0.407, slope—1; Fig 3B) and DCA curves (Fig 3C) of the nomogram respectively showed good fitting and clinical efficacy in the verification group.

### Comparing to other prediction methods

**Fig 4:**
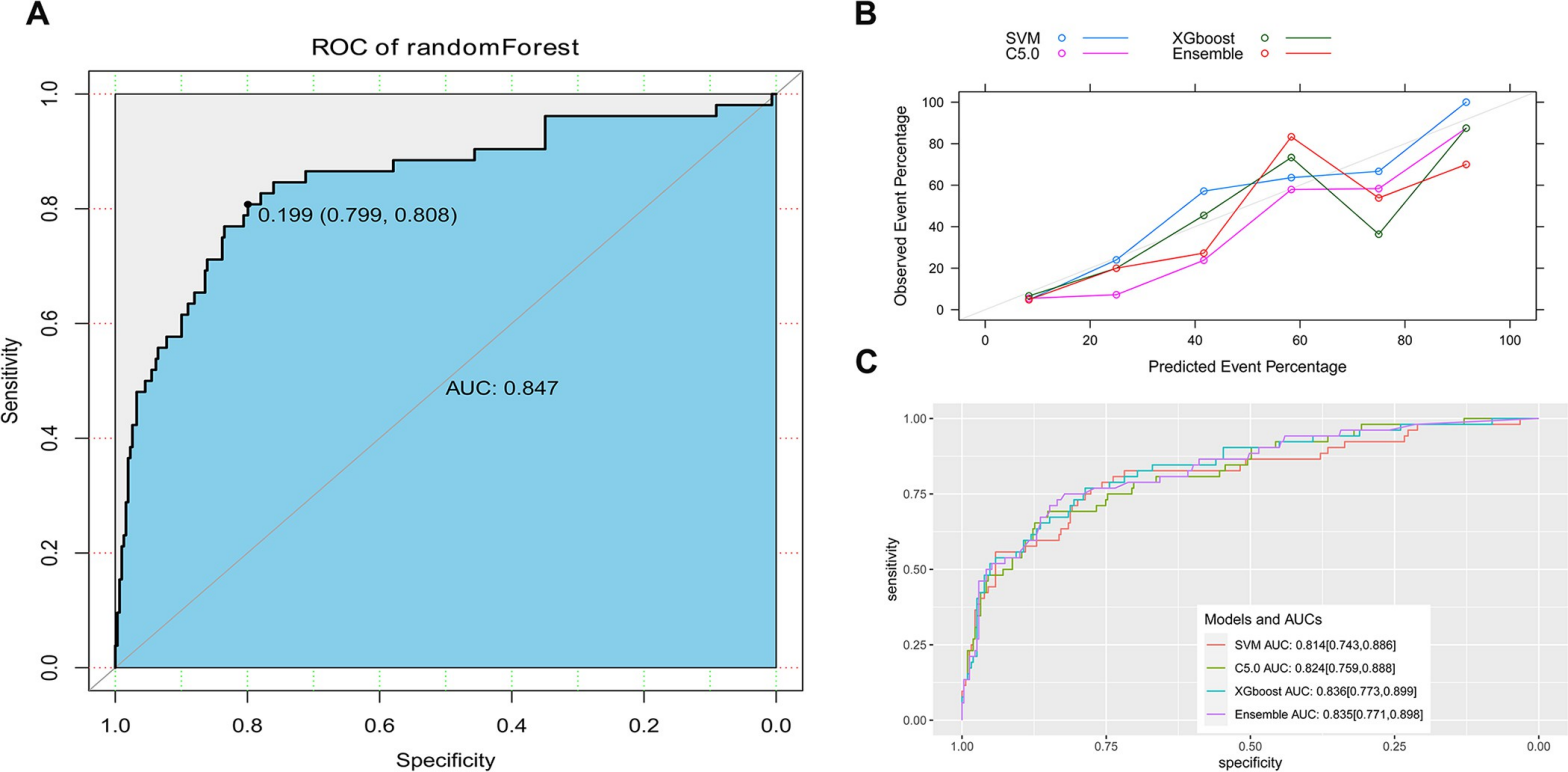
Evaluation of power in the validation population with other machine learning models. A, ROC curve of the model by Random Forest method; B, calibration chart of the model by Stacking method; C, ROC curve of the model by Stacking method.

The AUC value of the random forests modeling was 0.847 in the validation group (Fig 4A), which was comparable to the established logistic prediction model (P = 0.430). In the stacking method, three methods (SVM, support vector machine; XGBoost, extreme gradient boosting; C 5.0) were chosen. The calibration of the final integrated model is poor in the validation group (Fig 4B). The AUC value of the final integrated model was 0.835 in the validation group, which was comparable to the established logistic prediction model (P = 0.280) (Fig 4C).

**Fig 5:**
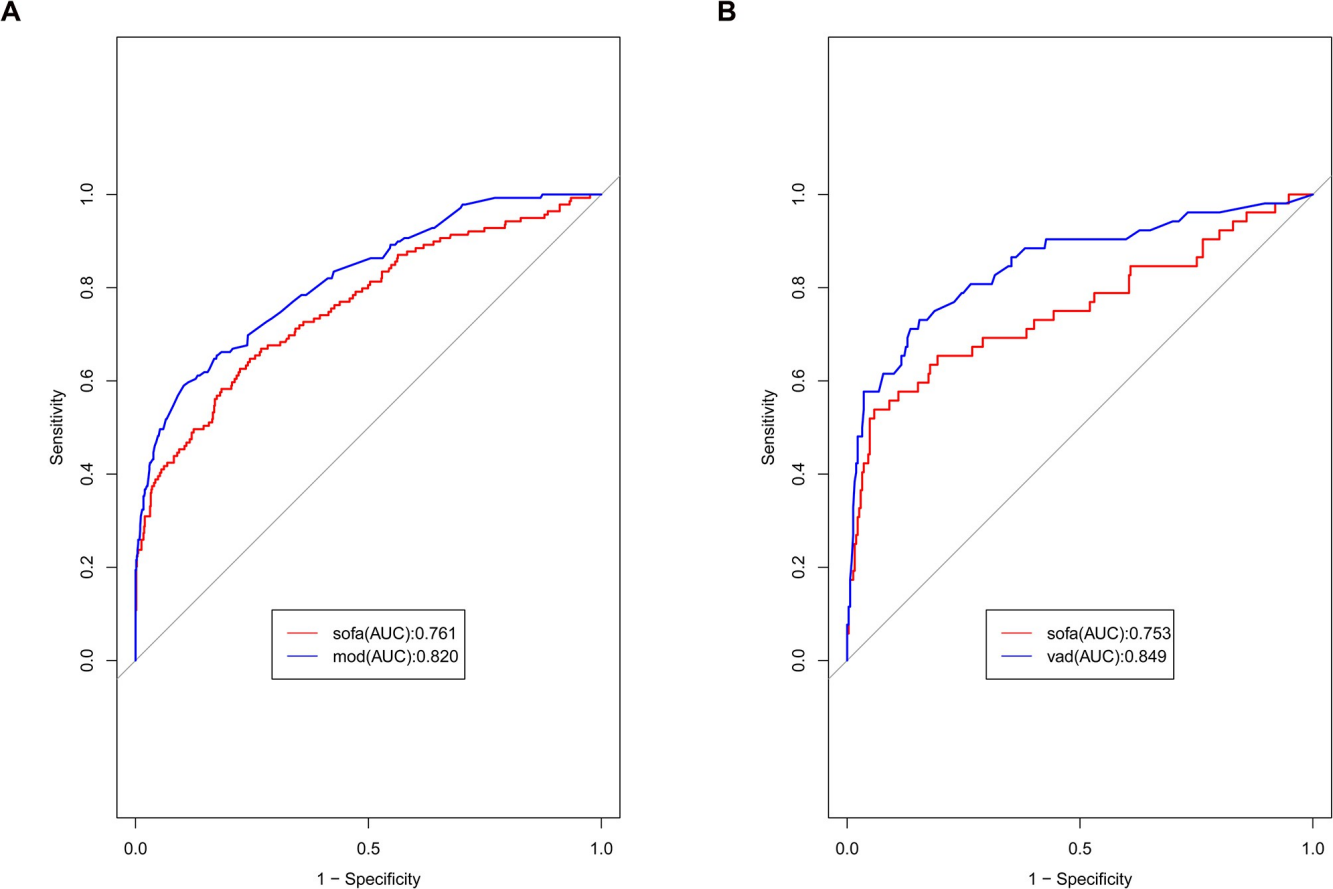
Comparison of ROCs for models based on the logistic regression and SOFA. A, ROC comparison in training group; B, ROC comparison in validation group.

Based on the SOFA scoring system, the AUC of the prediction model in the training group was 0.761 (Fig 5A), which was significantly lower than that of established nomogram prediction model (*P* = 0.008, Delong test). The AUC of the prediction model in the validation group was 0.753 (Fig 5B), which was significantly lower than that of established nomogram prediction model (*P* = 0.022).

## Discussion

Sepsis is the result of a disproportionate immune response to infection, which is unable to eliminate invading pathogens [30, 31] and manifests as multiple organ dysfunction entailing coagulation disorder, cardiac dysfunction, renal insufficiency, nutritional disorder etc. We established a prediction model for 30-day mortality in sepsis patients on the basis of pro-bnp, lactic acid, hct,and map at admission. The discriminatory power, calibration and clinical efficacy of the nomogram were significant, which indicates its potential for identifying sepsis patients at high risk of death in order to initiate intervention measures at the earliest.

Lactic acid is an indicator of oxygen metabolism and is used to gauge the prognosis in sepsis and other critical diseases [32–34]. Pro-BNP is an indicator of cardiac function and is relevant in sepsis since the latter can trigger septic cardiomyopathy. One study showed that the death rate is higher in sepsis patients with septic cardiomyopathy [35]. Moreover, inappropriate rehydration during treatment can also increase pro-BNP levels. Regardless of the cause, an aberrant increase in pro-BNP is an indicator of poor prognosis in sepsis patients [36].

The low MAP and high heart rate in sepsis patients are indicative of low perfusion shock and severe stage of the disease. Studies show that the mortality rate of septic shock is 33.5%-61% [37, 38], which significantly increases the risk of death in patients with sepsis. Many studies have explored the relationship between sepsis and acute respiratory distress syndrome (ARDS) [18], and have reported that ARDS increases the mortality rate of sepsis patients [39]. Nevertheless, little is known regarding the relationship between sepsis and oxygenation index. We found that the early decline in the oxygenation index is an independent risk factor for sepsis. In addition, ARDS, cardiac dysfunction and excessive fluid resuscitation can decrease the oxygenation index, and should be actively prevented and treated in the sepsis patients. Finally, we observed that the HCT value is predictive of the short-term prognosis of sepsis patients. Sub-normal HCT was associated with worse prognosis, suggesting that treating anemia can improve the prognosis of sepsis patients.

Various scoring systems are used to predict the prognosis of sepsis patients, such as the APACHEII and SOFA scoring systems, although they are fraught with lower predictive accuracy and reliability, along with insufficient discrimination power [8, 34, 40]. In addition, four scoring systems were evaluated by Arabi et al (including APACHEII and SOFA) in ICU patients with sepsis, all of which showed poor calibration [10]. Moreover, the clinical relevance of these scoring systems has not been evaluated previously [41]. In this study, the nomogram prediction model was superior than SOFA system regarding on the discrimination power (AUC), which could be partly explained by the difference among the enrolled variables of these two groups. Furthermore, the predictive factors included in this study are objective and simple and conveniently obtained after admission, indicating potential clinical efficacy. Predicting the death risks after admission could help clinical physicians to make effective therapeutic regimens with good prognosis.

This nomogram prediction model was comparable to those based on random forests model and stacking model regarding on the discrimination power. The stacking method integrates three models of SVM, C5.0 and Xgboost. The nomogram graph, based on logistic regression model, could visually show how much the enrolled variables contributed to the predicted risk. For random forest method and stacking method, how the enrolled variables contributed to the predicted event remained unclear, and some showed little improvement to the performance of prediction model, resulting into less practice. Only when the increased complexity leads to significant improvement in the efficiency of a model, it is reasonable to add complex algorithms to establish a model [42]. Our nomogram model owned comparable AUCs to models based on the stacking method, with less variables enrolled and more feasibility.

Nevertheless, there are some limitations in our study that ought to be considered. First, as a retrospective study, the selection bias could not be avoided as patients with missing enrolled variables were excluded. Second, it was a single-center study with limited number of cases, and a real external validation dataset was lacking. Third, this established model could not be compared to APACHE II scoring system due to unavailable data from clinical record. Moreover, other potential risk factors such as comorbidities, were not considered in this study. These limitations might reduce the prediction ability of established model in another patient population.

## Conclusion

A novel nomogram based on multiple clinical indices was constructed that can predict the short-term prognosis of sepsis patients accurately and reliably, which will be helpful for clinical decision-making.

## Supporting information

S1 Checklist STROBE 2007 (v4) checklist of items to be included in reports of observational studies in epidemiology*. (PDF)

S1 Fig The flowchart for patient inclusion and exclusion. (TIF)

S1 Table The interaction of variables in training group. (PDF)

S2 Table Vif of variables in training group. (PDF)

S1 File Raw data. (CSV)
