# Supplementary material for: A nomogram for predicting mortality risk within 30 days in sepsis patients admitted in the emergency department: A retrospective analysis
Source: PLoS One. 2024 Jan 25;19(1):e0296456. doi: 10.1371/journal.pone.0296456 (PMC10810512; doi:10.1371/journal.pone.0296456)
Supplement: S1 Table — (PDF) [file pone.0296456.s003.pdf]

S1 table. The interaction of Variables in modeling group.

Variables	Estimate	Std. Error	z value	Pr(>|z|)
(PaO2/FiO2)<200:creatinine125-350	-0.27640	0.60911	-0.454	0.649990
(PaO2/FiO2)201-300:creatinine125-350	-0.69079	0.58493	-1.181	0.237613
(PaO2/FiO2)<200:creatinine>350	-0.29225	1.05850	-0.276	0.782472
(PaO2/FiO2)201-300:creatinine>350	-1.45007	0.92441	-1.569	0.116732
(PaO2/FiO2)<200:albumin<25	-2.105941	1.420653	-1.482	0.138241
(PaO2/FiO2)201-300:albumin<25	-0.598462	1.644459	-0.364	0.715913
(PaO2/FiO2)<200:albumin25-35	-1.009524	1.407132	-0.717	0.473107
(PaO2/FiO2)201-300:albumin25-35	0.427307	1.641425	0.260	0.794612
(PaO2/FiO2)<200:map<60	0.5700	1.4011	0.407	0.684135
(PaO2/FiO2)201-300:map<60	1.6690	1.4543	1.148	0.251096
(PaO2/FiO2)<200:map>90	-0.1438	0.5782	-0.249	0.803549
(PaO2/FiO2)201-300:map>90	-0.5451	0.5752	-0.948	0.343303
(PaO2/FiO2)<200:hematocrit<0.3	0.3624	0.6073	0.597	0.55075
(PaO2/FiO2)201-300:hematocrit<0.3	-0.4999	0.5828	-0.858	0.39105
(PaO2/FiO2)<200:hematocrit>0.4	-0.6354	1.1575	-0.549	0.58304
(PaO2/FiO2)201-300:hematocrit>0.4	-0.3033	1.0795	-0.281	0.77873
(PaO2/FiO2)<200:pro.bnp 500-15000	-15.05154	741.91351	-0.020	0.983814
(PaO2/FiO2)201-300:pro.bnp 500-15000	-14.89846	741.91349	-0.020	0.983979
(PaO2/FiO2)<200:pro.bnp >15000	-15.05821	741.91371	-0.020	0.983807
(PaO2/FiO2)201-300:pro.bnp >15000	-15.91334	741.91372	-0.021	0.982887
(PaO2/FiO2)<200:lac4-10	-0.73395	0.60334	-1.216	0.223802
(PaO2/FiO2)201-300:lac4-10	-0.60516	0.57824	-1.047	0.295307
(PaO2/FiO2)<200:lac>10	-1.02123	1.12024	-0.912	0.361973
(PaO2/FiO2)201-300:lac>10	-1.83615	1.05818	-1.735	0.082704
creatinine125-350:albumin<25	-0.63470	1.26878	-0.500	0.616902
creatinine>350:albumin<25	-0.57025	1.81724	-0.314	0.753673
creatinine125-350:albumin25-35	-0.18513	1.24797	-0.148	0.882070
creatinine>350:albumin25-35	-1.25653	1.82579	-0.688	0.491320
creatinine125-350:map<60	0.6176	1.1547	0.535	0.592731
creatinine>350:map<60	11.9792	563.1501	0.021	0.983029
creatinine125-350:map>90	-0.1348	0.5007	-0.269	0.787829
creatinine>350:map>90	-0.3084	0.8150	-0.378	0.705184
creatinine125-350:hematocrit<0.3	0.32913	0.51030	0.645	0.518944
creatinine>350:hematocrit<0.3	0.05293	0.84610	0.063	0.950116
creatinine125-350:hematocrit>0.4	0.13709	0.92851	0.148	0.882622
creatinine>350:hematocrit>0.4	-11.51137	535.41195	-0.022	0.982847
creatinine125-350:pro.bnp 500-15000	-0.6449	1.1528	-0.559	0.575860
creatinine>350:pro.bnp 500-15000	10.6282	535.4119	0.020	0.984163
creatinine125-350:pro.bnp >15000	0.4372	1.4116	0.310	0.756775
creatinine>350:pro.bnp >15000	10.5077	535.4125	0.020	0.984342
creatinine125-350:lac4-10	-0.17836	0.51527	-0.346	0.729227
creatinine>350:lac4-10	-1.88006	0.98891	-1.901	0.057283

creatinine125-350:lac>10	-1.34581	0.92026	-1.462	0.143626
creatinine>350:lac>10	-1.97929	1.31558	-1.504	0.132453
albumin<25:map<60	-14.43428	590.93419	-0.024	0.98051
albumin25-35:map<60	-12.67849	590.93395	-0.021	0.98288
albumin<25:map>90	1.54414	1.36487	1.131	0.25791
albumin25-35:map>90	1.25360	1.35171	0.927	0.35371
albumin<25:hematocrit<0.3	0.84238	1.56003	0.540	0.589212
albumin25-35:hematocrit<0.3	0.78226	1.54080	0.508	0.611664
albumin<25:hematocrit>0.4	-0.73040	1.63662	-0.446	0.655393
albumin25-35:hematocrit>0.4	-1.25107	1.40492	-0.890	0.373202
albumin<25:pro.bnp 500-15000	15.57503	808.46006	0.019	0.984630
albumin25-35:pro.bnp 500-15000	0.45358	1.33966	0.339	0.734926
albumin<25:pro.bnp >15000	14.68766	808.46115	0.018	0.985505
albumin25-35:pro.bnp >15000	0.11145	1.87148	0.060	0.952514
albumin<25:lac4-10	0.3397	1.2237	0.278	0.781299
albumin25-35:lac4-10	-0.2384	1.2026	-0.198	0.842871
albumin<25:lac>10	0.2294	1.5333	0.150	0.881064
albumin25-35:lac>10	0.8076	1.5238	0.530	0.596114
map<60:hematocrit<0.3	1.0837	1.3071	0.829	0.407054
map>90:hematocrit<0.3	0.6065	0.5009	1.211	0.225894
map<60:hematocrit>0.4	14.2373	742.9953	0.019	0.984712
map>90:hematocrit>0.4	0.2835	0.9516	0.298	0.765793
map<60:pro.bnp 500-15000	-16.9921	1664.0396	-0.010	0.991853
map>90:pro.bnp 500-15000	0.2452	1.2916	0.190	0.849434
map<60:pro.bnp >15000	-2.8879	1802.3390	-0.002	0.998722
map>90:pro.bnp >15000	1.0460	1.3882	0.753	0.451170
map<60:lac4-10	1.1817	1.2111	0.976	0.329181
map>90:lac4-10	-0.3288	0.5131	-0.641	0.521710
map<60:lac>10	13.5341	629.5250	0.021	0.982848
map>90:lac>10	0.2343	0.8524	0.275	0.783368
hematocrit<0.3:pro.bnp500-15000	1.28037	1.24604	1.028	0.304161
hematocrit>0.4:pro.bnp 500-15000	1.60588	1.52700	1.052	0.292956
hematocrit<0.3:pro.bnp >15000	0.88678	1.33482	0.664	0.506471
hematocrit>0.4:pro.bnp >15000	-13.20059	620.52059	-0.021	0.983028
hematocrit<0.3:lac4-10	0.10743	0.50637	0.212	0.83199
hematocrit>0.4:lac4-10	-0.29743	0.90537	-0.329	0.74252
hematocrit<0.3:lac>10	0.81754	0.95067	0.860	0.38981
hematocrit>0.4:lac>10	-1.48085	1.50629	-0.983	0.32555
pro.bnp 500-15000:lac4-10	-0.83262	1.08370	-0.768	0.442301
pro.bnp >15000:lac4-10	-0.57774	1.20504	-0.479	0.631628
pro.bnp 500-15000:lac>10	14.91585	1687.77210	0.009	0.992949
pro.bnp >15000:lac>10	30.01338	1835.25588	0.016	0.986952

Callout:map, mean arterial pressure; pro.bnp, pro-brain natriuretic peptide; lac, lactic acid.
