# Supplementary material for: A nomogram for predicting mortality risk within 30 days in sepsis patients admitted in the emergency department: A retrospective analysis
Source: PLoS One. 2024 Jan 25;19(1):e0296456. doi: 10.1371/journal.pone.0296456 (PMC10810512; doi:10.1371/journal.pone.0296456)
Supplement: S2 Table — (PDF) [file pone.0296456.s004.pdf]

S2 table. Vif of variables in modeling group.

Variables	VIF
Albumin<25	4.177986
Albumin 25-35	4.067669
Pro.bnp>15000	1.947385
Pro.bnp 500-15000	1.737752
(PaO2/FiO2)<200	1.212399
Lac>10	1.178715
Creatinine 125-350	1.184765
Map<60	1.179941
Creatinine >350	1.174679
Hematocrit <0.3	1.126319
(PaO2/FiO2) 201-300	1.151869
Lac 4-10	1.121022
Hematocrit >0.4	1.069497
Map>90	1.044175

Callout: map, mean arterial pressure; pro.bnp, pro-brain natriuretic peptide; lac, lactic acid.
